# Role of Botulinum Toxin in Treatment of Secondary Dystonia: A Case Series and Overview of Literature

**DOI:** 10.3390/toxins16070286

**Published:** 2024-06-24

**Authors:** Diksha Mohanty, Heather R. M. Riordan, Peter Hedera

**Affiliations:** 1 Movement Disorder and Neuromodulation Center, Department of Neurology, Weill Institute for Neurosciences, University of California, San Francisco, CA 94158, USA; 2 Phelps Center for Cerebral Palsy and Developmental Medicine, Division of Pediatric Neurology, Kennedy Krieger Institute, Baltimore, MD 21205, USA; riordanh@kennedykrieger.org; 3 Division of Movement Disorders, Department of Neurology, University of Louisville School of Medicine, Louisville, KY 40202, USA

**Keywords:** dystonia, neurodegeneration, botulinum toxin, secondary dystonia, heredodegenerative

## Abstract

Introduction: Dystonia can present in primary and secondary forms, depending on co-occurring symptoms and syndromic associations. In contrast to primary dystonia, secondary forms of dystonia are often associated with lesions in the putamen or globus pallidus. Such disorders are commonly neurodegenerative or neurometabolic conditions which produce varied neurologic as well as systemic manifestations other than dystonia. Chemo-denervation with botulinum toxin has been successfully used for focal or segmental dystonia. However, studies evaluating the effect of BoNT therapy on patients with secondary dystonia are sparse, given the heterogeneity in etiology and presentation. Methods: We present a series of patients with secondary dystonia who were managed with botulinum toxin therapy. Patients included in this series had a confirmed neurometabolic cause of dystonia. Results: A total of 14 patients, with ages ranging from 17 to 36 years, with disorders including Wilson’s disease, pantothenate kinase-associated neurodegeneration (PKAN), Niemann–Pick disease type C (NPC), glutaric aciduria type 1, Sanfilippo syndrome (Mucopolysaccharidosis Type IIIb), and GM2 gangliosidosis (Sandhoff disease) are presented. Most patients experienced a mild to moderate improvement in treated dystonia with benefits ranging from 6 to 12 weeks, with the median length of the benefits lasting approximately eight weeks, without any significant adverse effects. Conclusion: Although the secondary causes of dystonia are complex and diverse, our presented data and the available reports of the use of botulinum toxin support the conclusion that chemo-denervation plays an important role in symptom alleviation.

## 1. Introduction

While multiple definitions of dystonia have existed previously, a 2013 consensus statement of the International Parkinson and Movement Disorder Society task force stated: “Dystonia is a movement disorder characterized by sustained or intermittent muscle contractions causing abnormal, often repetitive, movements, postures, or both. Dystonic movements are typically patterned, twisting, and may be tremulous. Dystonia is often initiated or worsened by voluntary action and associated with overflow muscle activation” [1]. This hyperkinetic movement disorder is usually worsened with voluntary action and frequently may be associated with a dystonic tremor. Patients often report a sensory trick or maneuver to alleviate dystonic symptoms, called “geste antagoniste”, a term coined by Meige and Feindel in 1902, and later thought to produce changes in tactile and proprioceptive sensory feedback, thereby altering motor performance in the region affected by dystonia [2]. Overflow dystonia, which involves the contiguous spread of antagonistic muscle contractions from the primary affected site, can be seen in some forms of dystonia. Another associated clinical phenomenon called “mirror dystonia” can occur where abnormal dystonic posturing of muscles is produced in the affected part of a body when performing a task using the opposite unaffected body part.

In its most severe presentations, dystonia may be generalized and involve several muscle groups throughout the body, producing the fixed abnormal posturing of limbs, head, and/or neck. In other cases, it may be focal, involving a singular region or group of muscles [1]. Commonly encountered focal dystonia syndromes include the involvement of neck muscles (cervical dystonia), oromandibular muscles, periocular muscles (blepharospasm), laryngeal muscles (spasmodic dysphonia), or muscles of a whole or part of an extremity (e.g., writer’s cramp or musician’s cramp). Segmental dystonia involves two or more contiguous regions of the body. Another form of dystonia called task-specific dystonia involves symptom expression exclusively with the repetitive performance of specific tasks or activities.

Many classification systems have been used to describe dystonia, for example, age of onset, distribution, etiology, progression, and variability [3]. Disorders that cause dystonia can be both neurodegenerative and non-neurodegenerative. Primary or idiopathic dystonia is reserved for dystonic syndromes where secondary causes with structural abnormalities have been ruled out [1].

Primary dystonia is thought to represent a group of disorders where dystonia is the predominant or only symptom, in the absence of other symptoms or another identifiable syndrome. However, this terminology has been inconsistently used in the past and its definition has evolved over time [4]. This group of disorders is generally not considered to be associated with neurodegeneration [5]. Idiopathic dystonia is mostly thought to be genetically-based and genes commonly known to cause dystonia include TOR1A (DYT1), THAP1 (DYT6), ANO3 (DYT24), GNAL (DYT25), COL6A3, and CIZ1. In recent years, it has been found that shared biological pathways lead to dystonia, by influencing dopamine signaling, mitochondrial function, heavy metal accumulation, and calcium homeostasis, among other processes [6].

The neuropathology underlying dystonia can be localized largely to the striatum in the basal ganglia. Circuitry involving the cortex and basal ganglia are impaired by abnormal neuronal transmission. Disorders of neurotransmitter homeostasis, owing to an imbalance between dopamine and acetylcholine, drive abnormal motor activity [7]. These circuits are further known to be influenced by cerebellar output to the cortex via the ventrolateral thalamus, which is also known to play a role in the aberrant network producing sensorimotor symptoms [8]. Other abnormal neurotransmitter activity implicated in various types of dystonia, according to animal studies, include a heightened sensitivity to opioid receptor activation, the imbalance of phosphodiesterase-10A, and aberrant adenosine signaling [9,10]. In addition, brainstem afferents to the cerebellum have been reported to be involved, along with a description of cases where dystonia occurred secondary to posterior fossa tumors or co-occurred with degenerative ataxic syndromes, where the primary pathology involved the cerebellum and brainstem structures [11].

In contrast to primary dystonia, secondary dystonia or non-primary dystonia is often associated with lesions in the putamen, globus pallidus, brainstem, or cerebellum. [12,13]. These disorders are commonly associated with neurodegeneration and neurometabolic conditions wherein dystonia occurs as part of a syndrome that produces other varied neurological and systemic manifestations. Some heredodegenerative disorders commonly known to cause dystonia are pantothenate kinase-associated neurodegeneration (PKAN), Niemann–Pick type C, Wilson’s disease (WD), glutaric aciduria type 1, neuro-acanthocytosis, neuro-ferritinopathy, GM2 gangliosidosis, HARP syndrome, and metachromatic leukodystrophy [14,15]. They can be often recognized by specific clues from clinical features. Eye movement disorders, sudden-onset dystonia with rapid progression, cranial-onset dystonia in childhood, prominent oromandibular dystonia, and hemi-dystonia can be pertinent clues to a secondary process [16,17].

Systemic disorders are also known to be associated with dystonia and include syphilis, Sjogren’s syndrome, tuberculosis, lupus, antiphospholipid antibody syndrome, and hypoparathyroidism. Exposure to toxins such as carbon monoxide, manganese, cyanide, methanol, disulfiram, carbon disulfide, and methanol have also been linked to the causation of dystonia [18]. However, the most common cause of secondary dystonia is associated with structural lesions. A study by Strader et al. from 2011 evaluated etiological and demographic differences between 58 patients with secondary dystonia compared to 162 patients with idiopathic dystonia and found that almost all secondary dystonia was lesion-induced (infarct, hemorrhage, trauma, and gunshot wound) [14]. Among structural lesions, some locations are known to produce specific types of dystonia. For instance, brainstem lesions have been associated with cranial dystonia, putaminal lesions associated with hemi-body involvement or limb dystonia, and thalamic dystonia have been associated with hand dystonia [19].

Patients with dystonia can report discomfort on a spectrum, with more severe forms associated with fixed posturing, debilitating pain, and long-term complications, such as contractures or orthopedic disorders. Dystonia can lead to ‘status dystonicus’ which is a life-threatening medical emergency [20]. Therefore, timely therapeutic intervention is vital for ameliorating discomfort and improving quality of life, as well as preventing extreme symptoms and long-term complications [21]. The treatment of dystonia has typically consisted of medication, administration of a neurotoxin, and surgical therapy [22]. Chemo-denervation with botulinum toxin (BoNT) has been successfully used for focal or segmental dystonia; however, it is considered impractical for use in generalized or hemi-dystonia, due to the need for a large volume of toxin for complete symptom alleviation. In cases where a large number of muscles are involved, specific targets are selected that contribute maximally to functional impairment, pain, or orthopedic complications [23]. This is done to avoid exceeding the maximum permissible dose of toxin for weight. The updated guidelines of the American Academy of Neurology recommend botulinum toxin A or B therapy as the first line of therapy for focal or segmental dystonia [24,25]. At the time of publication of this paper, five formulations of botulinum toxin are approved for use by the Food and Drug Administration in the United States for cervical dystonia. These are onabotulinumtoxinA, abobotulinumtoxinA, incobotulinumtoxinA, daxibotulinumtoxinA, and rimabotulinumtoxinB.

Most current recommendations include patients with primary or idiopathic dystonia. Studies evaluating the effect of BoNT therapy on patients with secondary dystonia are sparse given the heterogeneity in etiology. Here we present a case series of patients with secondary dystonia who were managed with botulinum toxin therapy (Table 1). We included only patients with proven neurometabolic causes of dystonia who had available data regarding the severity of dystonia, botulinum A or B doses, and therapeutic response. The diagnosis was confirmed by either biochemical testing and/or genetic testing confirming biallelic mutations in the causative genes. We evaluated the severity of dystonia in the treated body segments using the Burke–Fahn–Marsden dystonia scale, even though we did not generate total scores [26]. Severity factors for the eyes (blepharospasm), mouth (oromandibular dystonia), neck (cervical dystonia), arm, and leg (limb dystonia) were assessed using scoring from 0 to 4. Data on the degree and duration of benefit were collected from the caregivers using a scale ranging from “no improvement” to “marked improvement (complete resolution of dystonic symptoms)” in the treated body segment.

In this series, we present 14 patients, with ages ranging from 17 to 36 years, of whom nine were males and five females (Table 1). Our series includes five patients diagnosed with Wilson’s disease (WD), three with pantothenate kinase-associated neurodegeneration (PKAN), two with Niemann–Pick disease type C (NPC), two with glutaric aciduria type 1, one with Sanfilippo syndrome (Mucopolysaccharidosis Type IIIb), and one with GM2 gangliosidosis (Sandhoff disease). Treated regions affected by dystonia in these patients included cervical dystonia, oromandibular dystonia, blepharospasm, and limb dystonia, involving both upper and lower limbs. Four commercially available botulinum toxins were used. Seven patients were treated with onabotulinumtoxinA, including some who switched to another medication during the study, with doses ranging from 300 U to 600 U, six with 1500 U of abobotulinumtoxinA, two patients with incobotulinumtoxinA with doses ranging from 200 to 300 U, and one with 20,000 U of rimabotulinumtoxinB. Most patients experienced mild to moderate improvements in treated dystonia with benefits ranging from 6 to 12 weeks, with the median length of benefits lasting approximately eight weeks. All patients had an ongoing therapy with a toxin. The type of toxin used was switched during the period of observation for two patients only—in one instance, onabotulinumtoxinA was switched to abobotulinumtoxinA, and in another, onabotulinumtoxinA was switched to rimabotulinumtoxinB as severe sialorrhea was also present and amenable to BoNT. None of the patients experienced any significant adverse effects; however, eight patients had a percutaneous endoscopic gastrostomy (PEG) tube in situ which may have mitigated the presence of iatrogenic dysphagia. While we did not assay for neutralizing antibodies against botulinum toxins, clinically, we did not observe any marked loss of efficacy that would be suggestive of the development of neutralizing antibodies. We also discussed a possibility of deep brain stimulation therapy for these patients but none of the reported patients underwent DBS surgery. The most common reason for this was patient and caregiver preference for continuation of therapy with botulinum toxin. 

## 2. Discussion

We present our case series of patients diagnosed with WD, pantothenate kinase-associated neurodegeneration, Niemann–Pick C, Sanfilippo syndrome, glutaric aciduria type 1, and GM2 gangliosidosis, who benefited from chemodenervation with botulinum toxins A and B. Early-onset dystonia can be also seen in Rett (like)-syndromes (MECP, FOXG1, and GNB1), Westphal variant of Huntington disease, disorders of monoamine neurotransmitter metabolism (GCH1, AADC, and TH), GLUT1 deficiency, DYT-related to ADCY5, disorders of manganese metabolism with DYT-PARK (SLC30A10), galactosemia (GALT), biotinidase-deficiency (BTD), biotin-thiamine-responsive basal ganglia disease (SLC19A3), Leigh syndrome, and Mitochondrial Encephalo-myopathy with Lactic Acidosis and Stroke-like episodes (MELAS) [15]. Among later-onset neurological disorders, those that can present with dystonia also include neuro-acanthocytosis (VPS13A1), neurodegeneration with brain iron accumulation (NBIA), such as *PLA2G6*-associated neurodegeneration (PLAN), Leber Hereditary Optic Neuropathy (LHON), POLG-related disorders, dentato-rubro-pallidoluysian atrophy (ATN1), and GM1 gangliosidosis [15].

Dystonia is a common neurologic finding in 10–65% of all symptomatic WD patients [27]. Dystonic symptoms vary from focal and segmental to generalized dystonia. Segmental or focal dystonia in the craniofacial region is especially very symptomatic with severe oromandibular dystonia (OMD), dysarthria, risus sardonicus with a forced, often exaggerated smile and dysphagia with a complete loss of speech and inability to swallow. The clinical utility of this therapy was reported in a small series of six WD patients [28]. A case series of five patients with jaw-opening OMD in WD from Brazil showed mild improvements in Burke–Fahn–Marsden dystonia scores following treatment with botulinum toxin type A [29]. An improvement in lower extremity secondary dystonia symptoms was also reported in a small series of four WD patients [30]. Our data further support the benefits of botulinum toxin treatment of WD patients with blepharospasm, OMD, cervical dystonia, and limb dystonia in all our treated WD patients who had relatively severe dystonia associated with paradoxical worsening after chelation therapy [31].

Neurodegeneration with brain iron accumulation (NBIA) comprises a heterogeneous group of disorders and PKAN is the most common cause of NBIA [32]. Dystonia is almost always present in classical and atypical late onset PKAN. It can involve any body segment, but the most common is oromandibular dystonia and dysarthria [33]. The reported therapeutic use of botulinum toxin in PKAN is limited to a few case reports where mild to moderate benefits were reported [34,35]. We observed the best response after injections for oromandibular dystonia in these patients and the response in other treated segments was variable, but still found to be beneficial by the caregivers.

Niemann–Pick C is a lysosomal storage disorder caused by the autosomal recessive inheritance of mutations in NPC 1 or 2 genes [36]. The disease can be early-infantile, late-infantile, or juvenile, as well as adult-onset. In terms of neurologic manifestations, movement disorders such as myoclonus and dystonia commonly occur in this disorder, besides vertical supranuclear gaze palsy, cerebellar ataxia, behavioral changes, and cognitive dysfunction. Both focal and generalized forms of dystonia have been reported with this disorder [37]. We report two patients with childhood-onset Niemann–Pick type C, both with cervical dystonia, one who also had blepharospasm. Both patients showed a moderate improvement in dystonic symptoms with botulinum toxin A.

Glutaric aciduria type 1 (GA-1) is an inborn error of metabolism stemming from a deficiency in glutaryl-coenzyme A dehydrogenase, which leads to an accumulation of glutaric acid, 3-hydroxy glutaric acid, and glutaconic acid. Both these substances can be neurotoxic, thereby producing neurological symptoms such as dystonia, seizures, neuroimaging with frontotemporal atrophy, and Sylvian fissure enlargement, as well as caudate and putaminal hyperintensity [38]. Dystonia can occur both as a presenting feature or sequelae of disease. Treatment includes the administration of oral carnitine and riboflavin, alongside a protein-restricted diet, including a lysine-deficient special formulae. For the management of dystonia, trihexyphenidyl, intrathecal/intraventricular baclofen, and deep brain stimulation have been reported [39]. We report two patients with glutaric aciduria—one with dystonia of all extremities and another with cervical dystonia with upper extremity dystonia. In both these cases, the use of botulinum toxin A led to a mild improvement in symptoms, lasting about 8 weeks in each case.

GM2 gangliosidosis is an autosomal recessive lysosomal disorder caused by a biallelic mutation in HEXA or HEXB, which causes a deficiency of N-acetyl-P-hexosaminidase causing cognitive impairment, psychiatric symptoms, cerebellar ataxia, seizures, macular “cherry red” spots, lower motor neuron symptoms, and hepatosplenomegaly. Progressive focal, segmental, or generalized dystonia is a common presentation of the disorder [40]. The infantile and juvenile onset of the disease is more common than later onset [41]. We report one patient with GM2 gangliosidosis who presented with cervical and oromandibular dystonia as well as severe sialorrhea. Treatment with onabotulinumtoxinA yielded a mild improvement in OMD symptoms and a moderate improvement in cervical dystonia symptoms.

Additionally, we report one case of Mucopolysaccharidosis type IIIb, presenting with cervical and upper extremity dystonia, treated with onabotulinumtoxinA producing partial relief to dystonic symptoms in all segments. Subsequently, a more significant benefit was obtained from switching to rimabotulinumtoxinB, which also helped manage severe sialorrhea. This is also known as Sanfillipo syndrome and is caused by a defect in the metabolism of heparan sulfate. Besides dystonia, this disorder presents with developmental delay, seizures, frequent infections, intellectual disability, and loss of motor skills [42].

## 3. Conclusions

Secondary causes of dystonia are diverse and complex. Due to differences in etiological factors, presentation, involved regions, and disease progression, choice of treatment can vary. There is only limited evidence in the literature comparing available treatment modalities. Most of the reported patients experienced a mild to moderate improvement in their symptoms, but this was still judged to be meaningful, and all patients continued with botulinum toxin therapies. Our presented data and the available reports of the use of BoNT therapy support the conclusion that local chemo-denervation may play an important role in symptom alleviation in patients with secondary dystonia. It continues to be the mainstay of management in focal forms of dystonia, including secondary causes associated with widespread structural brain abnormalities.

## Figures and Tables

**Table 1:** Dystonia etiology, location, and response to botulinum toxin therapy.

Subject	Etiology and Age at Onset	Dystonia Pattern	Dystonia Severity	Botulinum Toxin Therapy	Outcome
Male, 27 years; 79 kg	Wilson’s disease; 18 years	Cervical dystonia, blepharospasm, oromandibular dystonia, dystonia of both upper extremities	Neck: 3 Mouth: 4 Eyes: 3 Right arm: 3 Left arm: 4	onabotulinumtoxinA 600 U (300 U CD, 80 U blepharospasm, 100 U OMD, 80 U R arm, 120 U Left arm × 3 cycles; abobotulinumtoxinA 1500 U (750 U CD, 50 U blepharospasm, 100 OMD, 200 U Right arm, 400 U Left arm × 8 cycles, ongoing therapy	onabotulinumtoxinA relief of dystonia for about 8 weeks; abobotulinumtoxinA relief of dystonia for about 10 weeks
Female, 22 years; 64 kg	Wilson’s disease; 19 years	Cervical dystonia, dystonia of left arm and left leg	Neck: 3 Left arm: 4 Left leg: 3	abobotulinumtoxinA 1500 U (500 U CD, 500 U each extremity) × 12 cycles, ongoing therapy	Improved CD, mild relief of limb dystonia lasting about 6 weeks
Female, 21 years; 74 kg	Wilson’s disease; 18 years	Oromandibular dystonia; severe sialorrhea also present	Mouth: 3	incobotulinumtoxinA 200 U (100 U OMD, 100 U sialorrhea) × 15 cycles, ongoing therapy	Improved OMD lasting 12 weeks
Male, 22 years; 87 kg	Wilson’s disease; 17 years	Cervical dystonia, blepharospasm, oromandibular dystonia	Neck: 3 Mouth: 3 Eyes: 3	onabotulinumtoxinA 500 U (300 U CD, 100 U blepharospasm, 100 U OMD, × 11 cycles	Improved CD, blepharospasm controlled for 11 weeks, mild relief of OMD lasting about 8 weeks
Male, 36 years; 91 kg	Wilson’s disease; 24 years	Dystonia of both upper and lower extremities	Right arm: 3 Left arm: 2 Right leg: 3 Left leg: 2	abobotulinumtoxinA 1500 U (500 U Right arm, 300 Right leg, 400 U arm, 300 U Left leg) × 10 cycles, ongoing therapy	Dystonia of all extremities improved for 9–10 weeks
Male, 18 years; 74 kg	PKAN (Pantothenate kinase-associated neurodegeneration); 13 years	Oromandibular dystonia, dystonia of both upper and lower extremities	Mouth: 3 Right arm: 3 Left arm: 3 Right leg: 3 Left leg: 3	onabotulinumtoxinA 600 U (100 U OMD, 200 U Right arm, 150 U Left arm, 75 U each leg) × 6 cycles	Mild and inconsistent improvement in all dystonic segments, lasting about 8 weeks
Male, 19 years; 70 kg	PKAN (Pantothenate kinase-associated neurodegeneration); 11 years	Cervical dystonia, oromandibular dystonia, dystonia of both upper extremities and left leg	Mouth: 3 Neck: 3 Right arm: 4 Left arm: 3 Left leg: 2	abobotulinumtoxinA 1500 U (500 U CD, 100 U OMD, 500 U Right arm, 200 U Right leg) × 9 cycles, ongoing therapy	Improved CD, blepharospasm controlled for 10 weeks, moderate improvement in OMD lasting about 8 weeks
Female, 17 years; 53 kg	PKAN (Pantothenate kinase-associated neurodegeneration); 7 years	Cervical dystonia, oromandibular dystonia, dystonia of both upper extremities	Mouth: 3 Neck: 3 Right arm: 4 Left arm: 4	abobotulinumtoxinA 1500 U (750 U CD, 150 U OMD, 300 U each arm) × 7 cycles	Improved CD, OMD for 10 weeks; mild improvement in arm dystonia lasting about 7–8 weeks
Male, 22 years; 79 kg	Niemann–Pick type C; 15 years	Cervical dystonia	Neck: 3	onabotulinumtoxinA 300 U (300 U CD) × 13 cycles, ongoing therapy	Marked improved CD for 11–12 weeks
Male, 31 years; 92 kg	Niemann–Pick type C; 19 years	Cervical dystonia, blepharospasm	Neck: 3 Eyes: 2	incobotulinumtoxinA 300 U (250 U CN, 50 U blepharospasm) × 14 cycles, ongoing therapy	Marked improved CD, blepharospasm for 11–12 weeks
Female, 28 years; 59 kg	Sanfilippo syndrome (Mucopolysaccharidosis Type IIIb); 4 years	Cervical dystonia, dystonia of both upper extremities, severe sialorrhea also present	Neck: 3 Right arm: 2 Left arm: 2	onabotulinumtoxinA 400 U (200 U CD, 100 U each arm) × 3 cycles rimabotulinumtoxinB 20,000 U (10,000 U CD, 5000 U sialorrhea, 5000 each arm) × 8 cycles, ongoing	onabotulinumtoxinA partial relief of dystonia for about 7 weeks; rimabotulinumtoxinB improved dystonia in every segment for 10 weeks
Male, 19 years; 71 kg	Glutaric aciduria type 1; 2 years	Dystonia of all four extremities	Right arm: 3 Left arm: 2 Right leg: 3 Left leg: 3	abobotulinumtoxinA 1500 U (500 U Right arm, 200 U Left arm, CD, 400 U each lower extremity) × 7 cycles, ongoing therapy	Mild improvement in dystonia in extremities for about 8–9 weeks
Female, 18 years; 61 kg	Glutaric aciduria type; 12 years	Cervical dystonia, dystonia of both arms	Neck: 3 Right arm: 3 Left arm: 3	onabotulinumtoxinA 600 U (200 U CD, 200 U each arm) × 11 cycles, ongoing therapy	Mild improvement in CD for 8 weeks; mild and inconsistent improvement in dystonia in extremities; dystonia lasting about 6–8 weeks
Male, 19 years; 54 kg	GM2 Gangliosidosis (Sandhoff disease); 9 years	Cervical dystonia, oromandibular dystonia, severe sialorrhea also present	Mouth: 3 Neck: 3	onabotulinumtoxinA 400 U (100 U OMD, 300 U CD) × 9 cycles, ongoing therapy	Mild improvement in OMD for 9 weeks; moderate improvement in CD for 10 weeks

## Data Availability

Data is contained within the article.
